# Editorial: The role of innate immunity in the pathogenesis of autoimmune and autoinflammatory diseases

**DOI:** 10.3389/fimmu.2026.1866180

**Published:** 2026-05-08

**Authors:** Carmelo Carmona-Rivera, Diana Gómez-Martín, José Luis Maravillas-Montero, José Jiram Torres-Ruiz

**Affiliations:** 1 National Institute of Arthritis and Musculoskeletal and Skin Diseases, National Institutes of Health, Bethesda, MA, United States; 2 Departamento de Inmunología y Reumatología, Instituto Nacional de Ciencias Médicas y Nutrición Salvador Zubirán, Mexico City, Mexico; 3 Departamento de Medicina Molecular y Bioprocesos, Instituto de Biotecnología, Universidad Nacional Autónoma de México, Cuernavaca, Mexico

**Keywords:** autoimmune diseases, autoinflammatory diseases, biomarkers, immune dysregulation, immunometabolism, innate immunity, trained immunity

Historically, research on autoimmune diseases has primarily emphasized adaptive immunity. However, this perspective is increasingly recognized as insufficient, as it does not fully capture the complexity and heterogeneity of immune-mediated disorders. The articles in this Research Topic collectively support a conceptual shift that positions innate immunity as a key player of disease initiation, amplification, and tissue injury. Despite employing diverse methodologies, including mechanistic studies, omics analyses, clinical investigations, and systematic reviews, all articles converge on the view that innate immune dysregulation is fundamental across autoimmune and autoinflammatory diseases.

A central theme of this Research Topic is the plasticity and heterogeneity of innate immune cells. Macrophages, dendritic cells, neutrophils, and innate lymphoid cells operate along dynamic activation spectra shaped by metabolic, epigenetic, and environmental cues. For example, Yan and Wan demonstrate that WTAP-mediated m6A modification regulates macrophage polarization in rheumatoid arthritis, linking post-transcriptional control to inflammatory phenotypes. Bai et al. identify CITED2 as a key hub gene associated with M1 macrophages in Hashimoto’s thyroiditis, underscoring the role of macrophage-driven inflammation in organ-specific autoimmunity. Similarly, Shao et al. show that NK cell exhaustion and interferon activation correlate with disease severity in anti-MDA5-positive dermatomyositis, highlighting the broader importance of innate lymphoid dysfunction, also seen in alopecia areata reviewed by Kim et al.

This Research Topic further examines the role of dendritic cells as key orchestrators bridging innate and adaptive immunity. Hara et al. describe the sequential activation of conventional and plasmacytoid dendritic cells, revealing both shared and disease-specific pathways in systemic lupus erythematosus and autoimmune pancreatitis. Prado et al. extend these insights by emphasizing the diverse roles of innate immune cells in multiple sclerosis, broadening the relevance of innate immune dysregulation beyond organ-specific diseases. Neutrophils and systemic inflammatory markers are also highlighted. Zhang et al. introduce the neutrophil-to-albumin ratio as a predictor of osteoporosis in rheumatoid arthritis, illustrating the clinical utility of innate immune-derived biomarkers for risk stratification.

Beyond cellular phenotypes, this Research Topic highlights the integration of innate immune signaling with metabolic and transcriptional reprogramming. Matmat et al. show that IFNγ-associated immunometabolic remodeling drives a serotonin-kynurenine imbalance, increasing cortical vulnerability in lupus-prone mice and underscoring the systemic impact of innate immune pathways. Xia et al. review lactylation as a central metabolic-immune interface, establishing metabolic intermediates as active regulators of the immune function rather than passive byproducts. The concept of trained immunity expands this framework: Salari et al. describe how epigenetic reprogramming of innate immune cells generates memory-like responses that can either enhance host defense or sustain chronic inflammation. This duality challenges the traditional distinction between innate and adaptive immunity and offers a unifying explanation for persistent inflammatory states.

Environmental and endogenous triggers also emerge as key modulators of innate immune activation. Tuncer et al. demonstrates that Epstein-Barr virus antigens induce anti-C1q autoantibodies that exacerbate lupus nephritis, exemplifying the interplay between infection and innate immune mechanisms. Similarly, Tong et al. show that pattern recognition receptors in pancreatic β cells contribute to disease pathogenesis, highlighting the role of non-immune cells in innate immune sensing.

A major strength of this Research Topic is the application of omics-based approaches to identify biomarkers and therapeutic targets. Wang et al. identify integrated stress response genes as biomarkers in ankylosing spondylitis, while Baltsiotis et al. propose STAT1 as a potential therapeutic target in thrombotic antiphospholipid syndrome. Alcalá-Carmona et al. further identify risk factors for fibromyalgia in idiopathic inflammatory myopathies. Collectively, these studies demonstrate the value of multidimensional approaches in capturing disease complexity.

Several contributions also address therapeutic implications. Vikár and Mocsai review tyrosine kinase signaling pathways as targets in pemphigoid diseases. Bai et al. provide comparative data on the risk of uveitis associated with JAK inhibitors versus TNF inhibitors in inflammatory conditions. Ahmed et al. review alopecia areata, linking its immunopathogenesis to emerging therapies and illustrating how mechanistic insights can inform clinical innovation. Gao et al. show that physical activity significantly influences outcomes in Sjögren’s syndrome, emphasizing the importance of non-pharmacological interventions in modulating immune responses.

Sierra-Salazar et al. broaden the scope of this Research Topic by examining adverse events related to cancer immunotherapy, demonstrating that innate immune mechanisms can also drive treatment-associated toxicity. Additional studies provide mechanistic insight into LACC1 (Li et al.), a gene increasingly implicated in autoinflammatory diseases. Mendes-Frías et al. show that type 1 diabetes alters inflammatory responses in periodontal disease, further illustrating systemic immune interactions.

This Research Topic also addresses the clinical spectrum of autoinflammatory diseases, including rare conditions such as mevalonic aciduria reported by Xue et al., highlighting the importance of early recognition and the impact of innate immune dysregulation in monogenic disorders.

Collectively, the articles in this Research Topic converge on a central concept: innate immune dysregulation links autoimmunity and autoinflammation along a continuum. This perspective challenges traditional classifications and supports a more integrated framework incorporating cellular plasticity, metabolic reprogramming, and environmental elements. Emerging technologies, including single-cell and spatial omics, will be essential to further resolve the complexity of innate immunity in human disease. Equally important is the translation of these insights into clinical practice to enable improved patient stratification, identification of novel therapeutic targets, and the development of personalized interventions based on innate immune signatures.

In conclusion, this Research Topic reflects a significant shift in immunology. Centering innate immunity in disease pathogenesis provides a conceptual and practical framework that may redefine the classification of autoimmune and autoinflammatory diseases. As Guest Editors, we hope this Research Topic will stimulate future research and accelerate the development of therapies targeting innate immune pathways.

